# Protocol for a community-engaged mixed-methods study for climate-related disaster preparedness and health care resilience in North Carolina

**DOI:** 10.3389/fpubh.2026.1805972

**Published:** 2026-04-16

**Authors:** Anna Tupetz, Nidhi Saripalli, Paige O'Leary, Grayson Chappell, Erin R. Hanlin

**Affiliations:** 1 Department of Emergency Medicine, Duke University School of Medicine, Durham, NC, United States; 2 Duke Global Health Institute, Duke University, Durham, NC, United States

**Keywords:** climate change, climate-related disasters, community-engaged, healthcare, mixed method, resilience

## Abstract

**Background:**

Climate change-induced disasters pose an enormous risk to human health. With the risk of climate change disasters at an all-time high, it is imperative to strengthen the resilience of health and community systems. Working with community collaborators who provide services across the spectrum of the disaster cycle will make disaster preparedness plans context-specific, sustainable, and accepted by the community. Using evidence-based Implementation Science approaches can facilitate the development and evaluation of community-academic partnership initiatives to build responsive and resilient systems.

**Objective:**

To identify perceived gaps and needs, available resources, and collaborator engagement patterns of community organizations and state agencies across the disaster cycle in North Carolina.

**Methods:**

This framework-guided state-wide project will first use Hurricane Helene as a case study to deploy an initial survey to all community partners and state agencies that provided services in the response and recovery efforts in Western North Carolina, followed by focus group discussions to identify available resources, gaps, and needs that can be addressed through sustained partnerships.

**Anticipated results:**

The resulting quantitative and qualitative data will be used to create a virtual resource map for the community, to identify existing gaps in disaster response, and to identify key collaborators working in the disaster response sector in North Carolina. These results will ultimately lead to the formation of a community-academic coalition (DukeNC4Health) that will develop a community-engaged disaster preparedness strategy grounded in practice, education, research, and policy.

## Introduction

Climate change is a major environmental concern that is no longer a distant threat but a present reality. Recently, climate change events have been increasingly frequent and severe. The United States has seen an increase in the number and severity of climate-driven disasters (1). These events, ranging from hurricanes to floods and wildfires, have profound consequences on human health. Disasters disrupt health systems, exacerbate chronic conditions and create acute injuries (2, 3). Their impact is amplified exponentially by the Social Determinants of Health, such as poverty, housing insecurity, unsanitary living conditions, lack of food and water and limited access to healthcare. The maximum brunt of this effect is borne by the underserved communities (4–6).

In North Carolina, the annual rate of climate-related disasters has tripled (1). One recent example is Hurricane Helene in September 2024, when Western North Carolina was hit with some of the most severe flooding the region has ever seen. It caused severe damage to life and property. Sweeping into the Appalachian regions of western North Carolina, Helene unleashed record-breaking rainfall, catastrophic flooding, and widespread mudslides, something that was not anticipated and left the people unprepared to deal with the consequences. The hurricane caused at least 108 deaths, with Buncombe County reporting the highest number of deaths at 42. It left millions without electricity, inflicted over $59.6 billion worth of damages, devastated about 822,000 acres of forest area and destroyed more than 125,000 homes. Entire communities were cut-off from healthcare, transportation, and communication. Hurricane Helene was the costliest disaster in North Carolina’s history (7). In the face of such disasters, it is important to have an adaptable healthcare and response system that can manage a sudden increase in resource demand, especially for marginalized populations.

The need for proactive solutions to potential climate change disasters is urgent given the rising disaster frequency and the compounding risks faced by underserved populations. Including community partners, first responders, and experts in evidence-based approaches is critical to build and sustain resilient communities and healthcare systems (6, 8). Implementation Science provides an evidence-based approach that is increasingly used in designing and evaluating disaster and humanitarian response and preparedness strategies (9–11). Although Implementation Science has increasingly been applied in disaster preparedness evaluations, most studies focus on its use after a disaster to evaluate preparedness rather than during the project planning phases (12). Utilizing Implementation Science approaches in the planning and design, we propose this framework-guided, multi-stage, community-engaged project, which aims to strengthen the resilience of health systems and communities in response to climate-related challenges in North Carolina, by (1) creating a statewide resource map of existing community based organizations active at any stage of the disaster cycle, (2) performing a gaps-and-needs assessment of the organizations and services included in the resource map to identify areas for collaboration and priority areas, and (3) building a community, state government, and academic partnership coalition to engage in a strategic exchange of knowledge and resources between community and academia.

## Materials and methods

**Figure 1:**
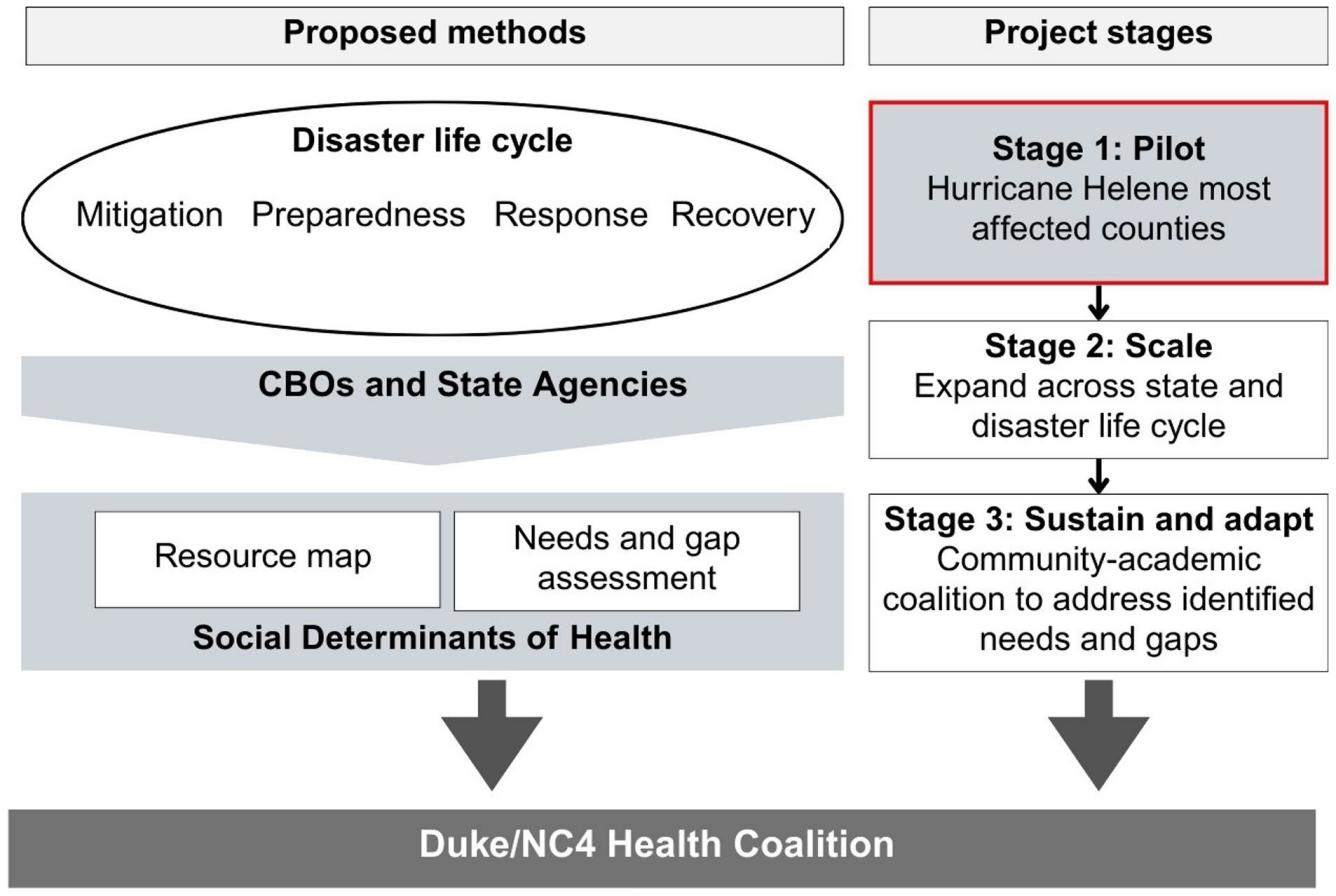
Project overview.

We will conduct a multi-stage, community-engaged, mixed-methods observational study, grounded in the Community Engaged Research Framework (CeRF) (13) and Implementation Science Frameworks. This study includes three stages: (1) Pilot, (2) Implementation, (3) Sustainment (Figure 1). This paper primarily focuses on the proposed methodology for the pilot stage.

### Stage 1: Pilot study

Stage 1 will serve as a proof-of-concept study to pilot our proposed study methods and anticipated end products and outcomes. We will focus on the most affected counties in North Carolina during Hurricane Helene in 2024. The strategy will be developed using two frameworks: (1) the Consolidated Framework for Implementation Research (CFIR2.0) (14) framework, and (2) the Re-AIM framework (15).

### Stage 2: Implementation

Revise the proposed strategy used in the pilot study and scale it across North Carolina (Figure 1) and throughout the disaster life cycle.

### Stage 3: Sustainment

Create a sustainable community-academic coalition for climate change disaster preparedness across NC. The mission of this coalition will be to address the identified gaps and needs by setting community-driven priorities, defining shared community goals, identifying future community-based academic projects to address these priorities and goals, and developing a disaster readiness plan for the community.

### Frameworks

Throughout all three stages of this project, we will use several research frameworks to guide our study methods.

#### Community-engaged research framework

CeRF is a framework consisting of 6 principles to apply throughout a research study when conducting community participatory research. The six principles are (1) Avoid harm, (2) Shared Decision making Power, (3) Transparency and Open Communication, (4) Accountability and Respect, (5) Accessibility and demonstrated Value, and (6) Capacity development and co-learning. The CeRF will be applied throughout our project, as we prioritize co-creation of deliverables, including the resource map and the coalition.

#### RE-AIM

RE-AIM is an implementation process framework widely used to guide the planning, design, or evaluation of Reach, Effectiveness, Adoption, Implementation, and Maintenance of an intervention (16). Therefore, RE-AIM will be used in this study for the planning, development, and implementation of our resource map and coalition creation. We will also use RE-AIM to guide our focus group discussion guides and codebook development to perform the gaps-and-needs analysis of available resources with collaborators.

#### Consolidated framework for implementation research

The CFIR is a determinant framework informed by implementation theories, models, and frameworks. CFIR is used to explore barriers and facilitators of implementation (17). CFIR 2.0 is an updated version of the original CFIR framework, based on multiple years of user feedback. CFIR 2.0 consists of constructs across 5 domains; (1) Innovation, (2) Outer setting, (3) Inner Setting, (4) Individuals: Roles and Characteristics, and (5) Implementation Process (14). CFIR 2.0 will be used in Stage 1 during the development of a community-engaged implementation strategy for the resource map. Specifically, components of CFIR 2.0 will be used to create the focus group discussion guide, and the codebook for the subsequent qualitative analysis.

#### Social determinants of health

The SDOH will serve as a framework to assess the social, economic, environmental, educational, and health care access components of disaster resources. These components of SDOH are known to impact health outcomes and disparities (4, 18). SDOH will guide the development and structure of the resource map.

### Ethical considerations

The presented study protocol has IRB approval from the Duke University Health System Institutional Review Board and was deemed exempt as study procedures pose a minimal level of risk. Informed consent will be obtained from participants, and confidentiality of data will be addressed by using unique identification numbers. We will use the STROBE (19) and COREQ (20) reporting guidelines when disseminating our findings.

### Study design

**Figure 2:**
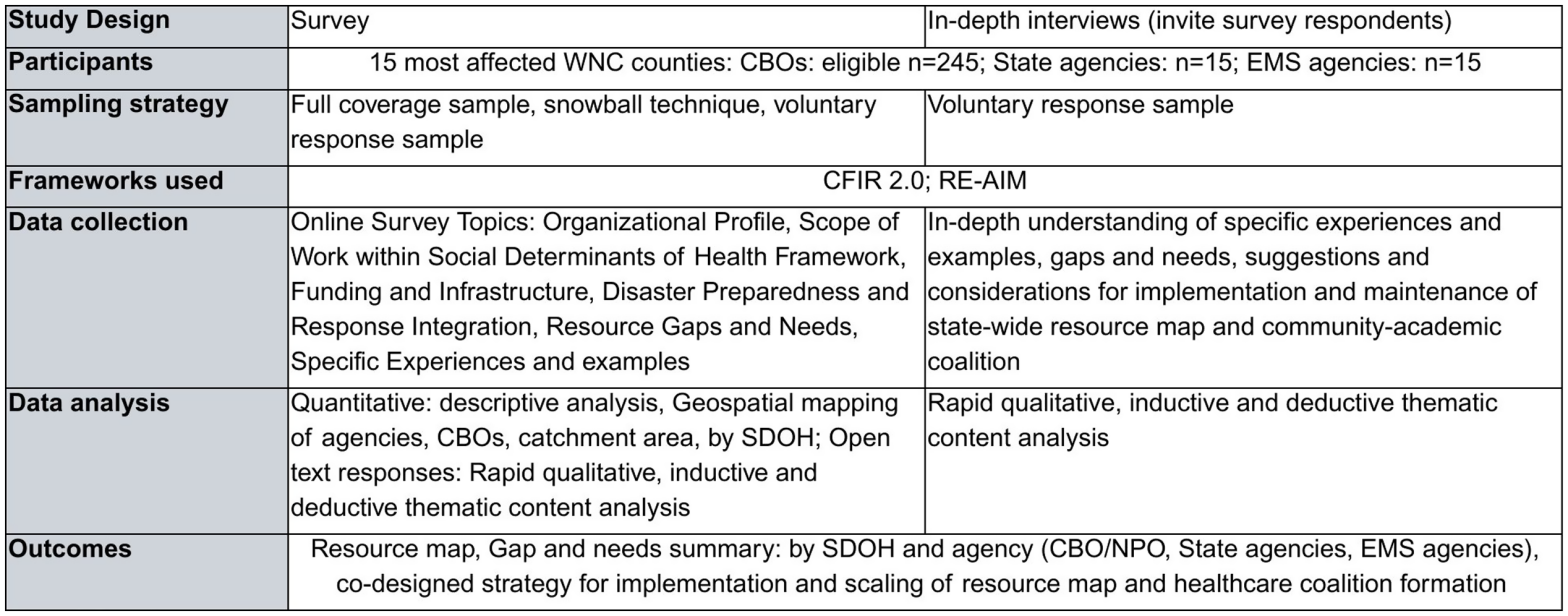
Stage 1 study methods.

This manuscript focuses on Stage 1 of the project, a proof-of-concept case study of Community-Based Organizations (CBO) and state agency response efforts to Hurricane Helene in Western North Carolina (Figure 2). Focusing on a limited number of counties that have recently experienced a climate-related disaster allows us to pilot and revise our methods before scaling the approach based on key interest holders’ input.

In this mixed-methods, community-engaged cross-sectional study using surveys and focus group discussions, we will identify available resources for the communities and perceived gaps and needs in response and recovery efforts following Hurricane Helene (Figure 2).

We will identify and connect with potential collaborators by creating a resource map, guided by the RE-AIM framework and grounded in tenets of the C-CAP process (Contextualize, Collect, Analyze, and Present) for community resource mapping (21). The resource map will be an interactive geospatial database of organizations (contact information, services provided, population served, etc.) that support the community during disaster response and recovery. The resource map will be organized using the Social Determinants of Health Framework by asking survey respondents to identify which social determinant of health their organization primarily serves. This will allow us to visualize existing resources, filter them by specific areas of interest, and identify social determinants that may be underrepresented in certain areas. We will further perform a gaps-and-needs analysis of existing CBOs and state agencies that participated in disaster response and recovery efforts after Hurricane Helene. Next, we will co-design a community engagement and implementation strategy for the resource map and healthcare coalition development. Following the principles of CeRF, newly identified collaborators in Stage 1 will be invited to remain engaged with the project and join a panel of existing partners with specific content expertise in disaster management and community-engaged research methods to cooperate on the planning and execution of Stages 2 and 3.

### Participants

We will include community and governmental organizations, Emergency Medical Services (EMS), and first responders who engaged in disaster relief during Hurricane Helene in the 15 most-affected counties of North Carolina. We are excluding organizations without a regular regional operational footprint and individuals not affiliated with any organization. Of the 39 North Carolina counties that received a federal disaster declaration, we will sample the 15 counties categorized by the North Carolina Office of State Budget and Management (OSBM) as suffering critical levels of business disruption. OSBM criteria for designating business disruption include FEMA designation and uptake of individual assistance (as a proxy for workforce disruption and displacement), duration and severity of electric power outages, search-and-rescue-based damage estimates as a proportion of county-assessed real property value, and the duration of county school closures (22). The following counties will be included in Stage 1: Ashe, Avery, Buncombe, Burke, Caldwell, Haywood, Henderson, Madison, McDowell, Mitchell, Polk, Rutherford, Transylvania, Watauga, and Yancey County.

### Sampling strategy

We will follow a full coverage sampling strategy and invite all eligible agencies and organizations to participate. We will identify all eligible initial CBOs by using the Guidestar’s online database for Non-profit and community-based organizations (23). This database of 1.9 million non-profit organizations allows to search by keywords, states, and counties. We will search for “Disaster” within the 15 most affected counties. This search yielded 245 results, all of whom will be invited to participate. Next, EMS (*n* = 15) and state agencies for disaster or emergency management (*n* = 15) are identified in each county via government websites. Specifically, we will identify the Emergency Management coordinators and EMS directors for each county. In addition to reviewing publicly available resources listed above, we will identify collaborators through the research team’s existing networks in emergency management and disaster preparedness, as well as through community-engagement research groups and conferences at their academic institutions. Following that initial step of identifying eligible groups (*n* = 275), this study will use a snowball/respondent-driven technique (non-probability sampling) to recruit participants through professional networks and during data collection. Each study participant will be asked to identify further resources and organizations that would be eligible to participate. Next, all survey respondents will be invited to participate in focus group discussions or in-depth interviews as scheduling permits.

### Data collection tools

#### Quantitative

We will develop a survey for CBOs, EMS, and state agencies to collect demographic information on organizational size, populations served, scope of activities according to the Social Determinants of Health Framework, languages spoken, catchment area, funding mechanisms, and integration into existing disaster preparedness and response networks. Next, we will ask them to identify gaps and needs within their organization to effectively pursue the organization’s mission, drawing on prior experience in their activities across the disaster life cycle and using the RE-AIM framework to guide the questions.

#### Qualitative

During in-depth interviews and focus group discussions, we will first invite participants to provide more in-depth explanations on identified gaps and needs of their organizations, as identified in open-text responses of the survey. Next, using tenets of the Re-AIM and CFIR framework, we will identify factors and suggestions to co-design and implement a state-wide resource map of available organizations, and ultimately, the formation of a climate disaster-focused community-state-academic healthcare coalition. Domains include Innovation, Outer setting, Inner setting, and Individuals of the CFIR framework. We will also discuss considerations for the implementation process and innovation/intervention outcomes, to create an effective and sustainable intervention. The Re-AIM framework will specifically ask about proposed strategies to reach the intended audience, factors that might affect the effectiveness of the proposed intervention, potential obstacles to maintaining engagement, and strategies to update and sustain the intervention. Intervention. Both the survey and focus group guides will be designed by the research team and piloted with EMS, state agency representatives, and CBOs from other states.

### Data collection

#### Quantitative

After informed electronic consent is obtained, electronic surveys will be sent to eligible participants either directly by the study team or, if participants already have academic collaborations, through those partnerships. Eligible participants and organizations will be approached up to three times to participate in this study. Quantitative data collection is complete once no new organizations and agencies are identified through the snowballing technique.

#### Qualitative

At the end of the survey, all participants will be asked to opt in to qualitative in-depth interviews or focus group discussions, which will be conducted either electronically or in person. Focus group discussions will be organized by region and audio-recorded. The discussions or in-depth interviews, based on participants’ preference, will be facilitated by a research member trained in qualitative data collection methods, with at least one additional note-taker present during data collection. The exact locations, dates, and number of focus group discussions will be determined by the number of survey participants who express interest. We anticipate focus groups of 5–10 participants per group, to allow for in-depth discussions. The focus group discussions or in-depth interviews will approximately last 45–90 min. Participants will be compensated for their time. We aim to perform focus group discussions and in-depth interviews until data saturation is reached. Research suggests that over 90% of themes emerge within the first 12 interviews (24). However, we expect differences in emerging codes across interest-holder groups (CBOs, State agencies, EMS groups), and therefore anticipate approximately 36–45 qualitative study participants. As we first approach agencies and community organization representatives whose operational language is English, data collection will be conducted in English.

### Data analysis

#### Quantitative

Survey responses that provide information on available resources will be analyzed descriptively and visualized as a spatial map. The final strategy for creating and implementing the resource map will be determined in collaboration with our Stage 1 partners. All statistical analyses will be conducted using RStudio. Quantitative survey data will be analyzed using descriptive statistics, including frequencies, percentages, and measures of central tendency, to characterize participant demographics, disaster exposure, and key outcome variables.

#### Qualitative

Qualitative data analysis of open-text responses from the survey on gaps and needs, and focus group/in-depth interviews, will follow a rapid qualitative analysis approach to summarize the feedback. We will apply the findings to the CFIR 2.0 and Re-AIM models to identify key domains to consider when planning and adapting an intervention for a specific setting. We will use rapid qualitative analysis to summarize the feedback, applied to CFIR2.0 and RE-AIM constructs, following a mix of deductive and inductive content analysis (25). A first codebook will be developed before data collection, which will include a coding matrix based on the CFIR and RE-AIM frameworks. After data collection and verbatim transcription of the audio recordings, two coders will create emerging codes (inductive) within each pre-defined theme (deductive). The developed codes will be compared and discussed in the case of discrepancies. If the discrepancies cannot be resolved, the principal investigator will make the final decision. Analysis will be completed using qualitative analysis software, such as Dedoose or Nvivo. Rapid analysis is appropriate and sufficient for research studies to inform primary study goals (26). Once analysis is completed, the qualitative findings will be shared with interview and focus group participants for member checking.

## Anticipated results

The planned main outcome of this study is the formation of a state-wide coalition (Duke/NC4Health) dedicated to advancing climate resilience through practice, policy, education, and research. This foundation will support future disaster implementation and evaluation efforts across North Carolina, thereby further improving global disaster-resilience standards.

### Stage 1: Pilot study

Through this pilot study, we aim to develop and refine a strategic community engagement and implementation plan to create, implement, disseminate, and maintain (1) a state-wide resource map to facilitate access to essential resources across the disaster life cycle, (2) a mixed-method gaps-and-needs assessment of the organizations and services included in the resource map, identifying opportunities for collaboration and priority areas, and (3) a sustainable community-academic coalition for climate change disaster preparedness across NC. The strategic plan will include (1) revisions of all the above-mentioned study methods, based on collaborators’ input and the study team’s assessment of identified challenges in study procedures, such as sampling strategy, recruitment rates, and data collection methods, and (2) a RE-AIM and CFIR guided strategy document to scale the proposed methods statewide. Furthermore, we will have formed new partnerships and deepened our existing partnerships with community and state agencies, who will be invited to continue our collaborations throughout Stages 2 and 3.

### Stage 2: Implementation

In Stage 2, the study methods that were piloted and refined in Stage 1 will be scaled to include all CBOs, EMS agencies, and emergency preparedness and disaster management state agencies across North Carolina working in all phases of the disaster life cycle to create a comprehensive resource map and gaps-and-needs assessment. The resource map will be used by community members, community organizations, EMS, and state agencies. The resource map will be translated into additional languages, as suggested by our collaborators, to increase accessibility. The gaps-and-needs assessment will be used primarily by the study team and collaborators to identify priority areas for future projects and activities. In Stage 2, collaborators will also provide suggestions and considerations to form and sustain the climate disaster-focused community-state-academic healthcare coalition in Stage 3.

### Stage 3: Sustainment

Based on findings and formed collaborations throughout stages 1 and 2, we will create a sustainable community-academic coalition for climate-change disaster preparedness across NC. This coalition will include representatives of CBOs, EMS, and state agencies to develop a community-engaged disaster preparedness strategy grounded in practice, education, research, and policy that serves and protects underserved communities, most impacted during climate change-related disasters.

## Discussion

This community-engaged mixed-methods study embedded in Implementation Science methodology is designed to address critical gaps in climate disaster preparedness in North Carolina by harnessing community experiences and leveraging cross-sector collaboration. The initial phase of this project, focusing on Hurricane Helene as a recent, high-impact climate event in North Carolina will serve as a proof of concept. After this initial phase, the project will be upscaled with aims to generate actionable insights into available disaster response resources and map how these resources are accessed and operationalized across diverse community populations throughout the State. The combination of quantitative resource mapping with qualitative inquiry allows for a nuanced understanding of community-level disaster resilience and is designed to generate practical, community-informed evidence to support more coordinated and equitable disaster preparedness.

The anticipated outputs of this study, including a comprehensive resource map, a strategic community engagement plan, and the establishment of a statewide climate and health coalition, have important implications for disaster preparedness, policy development, and future research. By identifying gaps in the existing disaster response infrastructure, creating new community-academic partnerships, and elevating community-defined priorities, this project has the potential to inform more equitable and effective disaster planning and research (27). Moreover, the methodological approach outlined here may serve as a replicable model for other regions facing increasing climate-related threats.

A key strength of this methodology is its grounding in established conceptual frameworks. The incorporation of implementation science principles, specifically CFIR and Re-AIM, facilitates the systematic identification of barriers and facilitators to effective disaster response and guides the planning, implementation, evaluation, and sustainment of a statewide intervention to improve disaster resilience (14, 15). Focus group discussions structured around CFIR constructs will enable collaborators to articulate the determinants that influence the organization’s activities in disaster response. Re-AIM methodology provides a framework for upscaling to a statewide resource map and for co-designing and implementing a feasible intervention to improve disaster resilience, namely the creation and sustainment of the Duke/NC4Health Coalition. Furthermore, the use of the SDoH framework ensures that available disaster response resources are examined through an equity lens, recognizing that climate-related health impacts are shaped by structural and social conditions such as housing stability, access to healthcare, transportation, and economic security (4, 28).

Community engagement is central to this study’s design and anticipated impact (29, 30). The snowball sampling approach is well-suited to identifying key informants and organizations involved in disaster response (31). By engaging community organizations, first responders, and governmental agencies as both participants and collaborators, this study prioritizes bidirectional knowledge exchange and seeks to build trust between academic institutions and communities. The planned formation of a community-academic coalition represents a critical step toward sustaining engagement beyond the study period and translating findings into long-term community-driven disaster readiness planning.

Several limitations should be considered. Snowball sampling as a type of non-probability sampling may introduce selection bias and limit the generalizability of findings; however, the goal of this study is depth and contextual relevance at the individual and organizational levels rather than population-level inferences (31). Additionally, reliance on self-reported data may be subject to recall bias, particularly given the traumatic nature of disaster experiences. However, iterative qualitative analyses will be performed, and interviews will be conducted until thematic saturation is achieved, ensuring adequate sample size and full exploration of core ideas.

In conclusion, this study addresses an urgent public health challenge by integrating community engagement, implementation science, and equity-focused frameworks to strengthen disaster resilience. As climate-related disasters continue to increase in frequency and severity, methodologies that foster community perspectives, promote cross-sector collaboration, and create scalable and sustainable interventions will be essential for protecting health and reducing disparities after disaster impacts.

## Data Availability

The original contributions presented in the study are included in the article/supplementary material, further inquiries can be directed to the corresponding author/s.
